# Grapevine leaf physiology and morphological characteristics to elevated CO_2_ in the VineyardFACE (Free air Carbon dioxide Enrichment) experiment

**DOI:** 10.3389/fpls.2022.1085878

**Published:** 2022-12-09

**Authors:** Yvette Wohlfahrt, Katja Krüger, Daniel Papsdorf, Susanne Tittmann, Manfred Stoll

**Affiliations:** ^1^ Department of General and Organic Viticulture, Hochschule Geisenheim University, Geisenheim, Germany; ^2^ University of Applied Sciences Erfurt, Erfurt Research Centre for Horticultural Crops (FGK), Erfurt, Germany; ^3^ Leibniz Institute of Vegetable and Ornamental Crops (IGZ), Erfurt, Germany; ^4^ Department of Applied Ecology, Hochschule Geisenheim University, Geisenheim, Germany

**Keywords:** leaf morphology, chlorophyll, *Vitis vinifera*, carbon dioxide, leaf physiology, histology, FACE (Free Air CO_2_ Enrichment)

## Abstract

Atmospheric carbon dioxide (CO_2_) concentration has continuously increased since pre-industrial times and has currently reached an average growth rate of 2.3 ppm per year. For the majority of plant species elevated CO_2_ (eCO_2_) improves photosynthesis and thus plant biomass production. To investigate the effects of eCO_2_ on leaf physiology and morphological leaf characteristics two *Vitis vinifera* L. cultivars, Riesling and Cabernet Sauvignon, grown in the VineyardFACE (Free Air Carbon dioxide Enrichment) system were used. The VineyardFACE is located at Geisenheim, Rheingau comparing future atmospheric CO_2_-concentrations (eCO_2_, predicted for the mid-21st century) with current ambient CO_2_-conditions (aCO_2_). Experiments were operated under rain-fed conditions for two consecutive years (2015 and 2016). For both varieties and CO_2_ treatments, leaf gas exchange measurements were performed as well as measures of epidermal flavonoid (Flav) and leaf chlorophyll (Chl) indices by using a portable leaf clip. Furthermore, leaves were sampled for spectrophotometric analysis of the leaf pigments chlorophyll a (Chl a), chlorophyll b (Chl b) and carotenoid (Car). Additionally, leaf cross-sections were produced as permanent preparations to investigate morphological characteristics of the leaf structure. Both cultivars did not differ in leaf chlorophyll meter readings or leaf pigments between the two CO_2_ treatments while net assimilation was highly stimulated under elevated CO_2_ for both seasons. Differences found in leaf cross-sections were detected in palisade parenchyma and epidermal thickness of Cabernet Sauvignon under eCO_2_, whereas Riesling net assimilation increased by 40% under a 20% CO_2_ enrichment while remaining unaffected in different leaf layer thickness. The observed results within grapevine leaf tissues provide insights to seasonal adaptation strategies of grapevines under elevated CO_2_ concentrations predicted in future.

## Introduction

Atmospheric carbon dioxide, one of the most relevant greenhouse gases has been increasing continuously since pre-industrial times from 280 ppm in 1750, and is predicted to exceed 700 ppm by the end of 21st century (IPCC, 2021). This accumulation of CO_2_ - among other air pollutants in the atmosphere - leads to a changed re-radiative effect and thus to an increase in global mean surface temperature - widely known as global warming. Besides that, high-pressure “blocking” weather systems (Davini and D’Andrea, 2020), an altered wind frequency and a shifting precipitation pattern are also consequences of a worldwide changing climate with an increasing intensity of extreme weather events (Manning and Tiedemann, 1995).

Plant and ecosystem performance is influenced by increasing CO_2_ levels leading to a modified plant physiology and thus to altered plant growth as well as developmental changes. For most of C_3_ plant species, elevated CO_2_ improves the photosynthetic apparatus resulting in an increased plant biomass production (Reddy et al., 2010) – in both – vegetative and reproductive performance. Besides agricultural crops, various CO_2_ enrichment experiments have been conducted worldwide for various plant types with CO_2_ effects on plant growth and ecosystems *via* a multitude of mechanisms (Ainsworth and Long, 2005). The up-regulation of photosynthesis under elevated CO_2_ as one main outcome is reported for most plant types. Likewise, water use efficiency, which is referred to net assimilation related to either transpiration or stomatal conductance, is shown to be improved under eCO_2_ conditions. Carbon metabolism in C_3_ plants is promoted under eCO_2_ due to higher carboxylation rates by RuBisCO and together with higher net assimilation rates are accountable for an enhanced biomass production.

Field studies on grapevines under elevated CO_2_ conditions that have been conducted are rare, showing higher yield and vegetative growth due to enhanced net assimilation rates (Bindi et al., 2001; Moutinho-Pereira et al., 2009; Edwards et al., 2017). Furthermore, in a previous study emerged from the VineyardFACE, Riesling and Cabernet Sauvignon resulted in higher lateral leaf area and leaf biomass, as well as increased bunch and berry weight under elevated CO_2_ concentrations (Wohlfahrt et al., 2018). Crop yield of Riesling showed a 10.4% (2015) and 17.8% (2016) increase under eCO_2_ and Cabernet Sauvignon gained 17.3% (2015) and 10.1% (2016) higher yield under eCO_2_. Effects on grapevine leaf transpiration and stomatal conductance are distinct, but most of the times the water demand decreased under eCO_2_ conditions when vines were mature at an age of 9 up to 20 years (Bindi et al., 2005; Tognetti et al., 2005; Moutinho-Pereira et al., 2009; Edwards et al., 2016; Edwards et al., 2017). Younger vines, at an age of 4 to 6 years showed a higher water consumption under eCO_2_ and therefore an increased leaf transpiration and stomatal conductance (Wohlfahrt et al., 2018). Nevertheless, independent of vine age, all previous studies observed an eCO_2_ effect on vine water use efficiency, which was shown to improve and has been supported by higher photosynthetic capacity under eCO_2_. As leaf photosynthesis occurs in chloroplasts of the mesophyll (palisade and spongy parenchyma) it is likely that an increased photosynthesis rate leads to an adaptation in morphological characteristics of leaves. Furthermore, spongy parenchyma has larger intercellular space for gas transportation, while palisade parenchyma is higher in chloroplast number and thus more beneficial to increase leaf photosynthesis.

Morphological alteration of leaves under eCO_2_ has been reported for several tree and agricultural C_3_ species, e.g. increase in leaf thickness and layers, extension of leaf cells and chloroplast development (Thomas and Harvey, 1983; Robertson and Leech, 1995; Saxe et al., 1998). The increase in leaf thickness of the grapevine cultivar Touriga Franca was derived from an extended spongy parenchyma and only partially due to an increase in palisade parenchyma under eCO_2_ conditions (Moutinho-Pereira et al., 2009).

The aim of this study was to investigate the effects of eCO_2_ on leaf physiology and morphological characteristics of the two *Vitis vinifera* L. cultivars Riesling and Cabernet Sauvignon grown in the VineyardFACE system and under temperate oceanic climate conditions.

## Material and methods

### Field site

The study was conducted at the VineyardFACE experimental site (49°59’N, 7°57’E) of Hochschule Geisenheim University, located in the Rheingau Valley, Germany, and was established as a ring system with six rings and a total area of 0.5 hectares. The vineyard used for the study was planted in 2012 using one-year-old pot-grown vines which were trained into a vertical shoot positioning system (VSP) and cane pruned to five nodes per square meter. Rows were north–south-orientated, while vine spacing was 0.9 m within rows and 1.8 m between rows. Two cultivars were used, *Vitis vinifera* L. cv. Riesling (clone 198–30 Gm) grafted on rootstock SO4 (clone 47 Gm) and cv. Cabernet Sauvignon (clone 170) grafted on rootstock 161–49 Couderc. Both rootstocks used are not considered to show a high tolerance against drought stress and were selected according to scion growth characteristics. Cultivars were bearing fruit for the first time in 2013, at an age of three years.

The soil at the field site is characterized as low-carbonate loamy sand to sandy loam with an average pH of 7.0 (0-30 cm, 30-60 cm, 60-90 cm). The available water capacity is 300 mm according to BFD5W (HLNUG, 2008). Management of vines was in accordance with the code of good practice (Bundesministerium für Ernährung Landwirtschaft und Verbraucherschutz - BMELV, 2010) and considered an Integrated Pest Management (IPM). Mineral fertilizer was amended with 50 kg N ha^-1^ a^-1^ before bloom (May). Cover crop consisted of Freudenberger WB 130 mulch mixture III, permanent vineyard greening I (Feldsaaten Freudenberger, Krefeld, Germany) in every second row, while every other row was ploughed. The cover crop mixture consisted of 10% perennial ryegrass, 20% Chewing’s fescue, 30% creeping red fescue and 40% Kentucky bluegrass and was mowed several times during vegetation. Shoot trimming was performed twice during vegetation, besides that no other canopy manipulation was conducted. Experiments were conducted under rain-fed conditions for two years, 2015 and 2016.

### VineyardFACE system and carbon dioxide treatments

For the simulation of an elevated atmospheric CO_2_ concentration, the VineyardFACE as a ring-shaped system started operating with a testing phase in 2013 comparing future atmospheric CO_2_-concentrations (eCO_2_) with current ambient CO_2_-conditions (aCO_2_). It is part of a special crop FACE system for permanent and annual crops implemented at Geisenheim University (**Supplementary Figure 1A**). Full operation of the still ongoing experiment started in 2014, including three ambient rings (aCO_2_) and three elevated rings (eCO_2_) with a targeted 20% CO_2_ increase in the eCO_2_ rings, which was the predicted concentration for 2050 (IPCC, 2014). Examples of an aCO_2_ and eCO_2_ ring during vegetation and the VineyardFACE experimental set-up are shown in **Supplementary Figure 1**. The VineyardFACE was described by Wohlfahrt et al. (2018) earlier. However, in brief each ring of the VineyardFACE system consisted of 36 jets, distributed in 10° steps, along a vertical double tubing system mounted at a height of 2.5 m, equipped with fans (MP25/4 T; CasaFan GmbH, Hasselroth, Germany) to create a high velocity downward air stream when activated and to allow a force-free pre-dilution of CO_2_. Real time measurements of wind direction and wind speed were used to determine the release of CO_2_ *via* transmitters (Thies Clima GmbH, Goettingen, Germany) installed in 3 m height. Depending on wind direction and wind speed fans operated in the upwind direction and only solenoid valve emitters on upwind-orientated side released CO_2_, unless wind speed was less than 0.1 m s^−1^ by Azimuth regulation (upwind control). The released CO_2_ was distributed throughout the ring by wind movement. Depending on the wind direction, the fans were switched on or off, with nine fans continuously on, covering a sector of 90° (**Supplementary Figure 2**). The CO_2_ release varied as a function of wind speed by adjusting the pulse-pause ratio of the CO_2_ releasing valves, the on time (pulse time) was fixed to 200 ms. According to the wind direction, five emitting valves were activated as shown in **Supplementary Figure 2**. No CO_2_ enrichment was carried out at wind speed < 0.1 m s^−1^ or air temperatures < 7°C. Fans in aCO_2_ rings were operated parallel to fans in eCO_2_ rings (E1-A1, E2-A2 and E3-A3) and where therefore defined as blocks. The data was recorded by a datalogger (CR800, Campbell Scientific, Logan, Utah, USA). Fumigation of CO_2_ was maintained during the entire year and from sunrise to sunset - mathematically calculated for the location of Geisenheim, Germany. To validate CO_2_ distribution within FACE rings, CO_2_ concentrations were recorded during an intensive period of monitoring in July 2015 using an infrared gas analyser (Li-Cor LI-8100CO_2_/H_2_O Analyzer and LI-8150 Multiplexer, Li-Cor Biosciences, Lincoln, NE, USA) at two different heights (0.8 and 1.7 m). Monitoring of the period from 14th to 22nd of July in 2015 is shown in **Supplementary Figure 3**. In 0.8 m height eCO_2_ concentration was 476 ppm, whereas aCO_2_ concentration remained at 397 ppm. At 1.7 m, CO_2_ concentration measured was 395 ppm for aCO_2_ and 458 ppm for eCO_2_. Whereas CO_2_ enrichment at 0.8 m was at the target of 20%, the CO_2_ enrichment concentration in 1.7 m was at 16%.

### Weather conditions

The climatic conditions are characterized by a temperate oceanic climate (Köppen-Geiger climate classification: Cfb (C-mild temperate, f-fully humid, b-warm summer); Chen and Chen, 2013) with mild winters and warm summers represented by an average annual temperature of 11.0°C (long-term average from 1991 to 2020) and mean annual rainfall of 527 mm. Mean daily temperature and precipitation data were collected from a weather station within the VineyardFACE. Precipitation and air temperature for the seasons 2015 and 2016 are shown in **Supplementary Figure 4**. Average growing season (1 April to 31 October) temperature was 15.9°C in both years, accumulated precipitation during the same time was 227 mm and 371 mm, in 2015 and 2016, respectively.

### Leaf gas exchange measurements

Leaf gas exchange measurements were conducted by using a portable open gas exchange system (GFS-3000, Walz, Effeltrich, Germany) to detect net assimilation rate (A). Measurements were performed on fully developed and physiological active, sun-exposed leaves on high solar irradiation days between 9 am to 1 pm at five or six time points per season. On each date three leaves of three vines per FACE-ring were measured. An external LED light source (1200 μmolm^−2^ s^−1^) was used which represented the mean light intensity of the measuring period. A 10-Liter buffer container was used for each of the two CO_2_ treatments to sample air within the rings by air intake of the gas analyser and to buffer short-term CO_2_ fluctuations. The carbon dioxide concentrations (CO_2 abs_) of the gas analyser was set to ambient to enable realistic CO_2_ conditions present in the field.

### Optical measurements

In both growing seasons, six mature primary leaves of six different vines per FACE-ring were measured on the adaxial and abaxial side with a Dualex Scientific portable optical leaf clip meter (Force A, Orsay, France) to determine epidermal flavonols (Flav) and leaf chlorophyll (Chl) indices according to Cerovic et al. (2012). Additionally, a nitrogen balance index (NBI) was calculated as the ratio of Chl and Flav. After execution of field measurement (02/09/2015 and 30/08/2016) same leaves were sampled to analyse leaf pigments.

### Leaf pigment analyses

Following optical measurements leaf samples were collected in black tubes and immediately frozen in liquid nitrogen in the field. Until further processing samples were stored at -80° C. Subsequently, leaves were grinded with pestle and mortar using liquid nitrogen under dark conditions to avoid damaging of pigments. Then samples were freeze-dried through the application of lyophilisation. For further analysis, 30 mg of freeze-dried sample were weighed in a 2 ml reaction tube with a spatula tip of sodium bicarbonate. The samples were extracted with 700 ml 100% aceton on ice for half an hour, mixed using a vortex (Reac control, Heidolph Instruments GmbH & Co. KG, Schwabach, Germany) and centrifuged at 4° C at 13.800 rpm (MiniSpin^®^ plus, Eppendorf SE, Hamburg, Germany). This washing step was repeated seven times. The supernatant was filtered using a syringe filter (0.45 µm) and 1 ml (10fold dilution) was transferred in a quartz cuvette (1 mm) for photometric analysis. The absorption at 400 to 780 nm was measured using a UV/Vis spectrophotometer (Specord 50, Analytik Jena GmbH, Jena, Germany). Chlorophyll a (Chl a), chlorophyll b (Chl b) and carotenoid (Car) content were determined according to Lichtenthaler (1987).

### Leaf histological analyses

**Figure 1:**
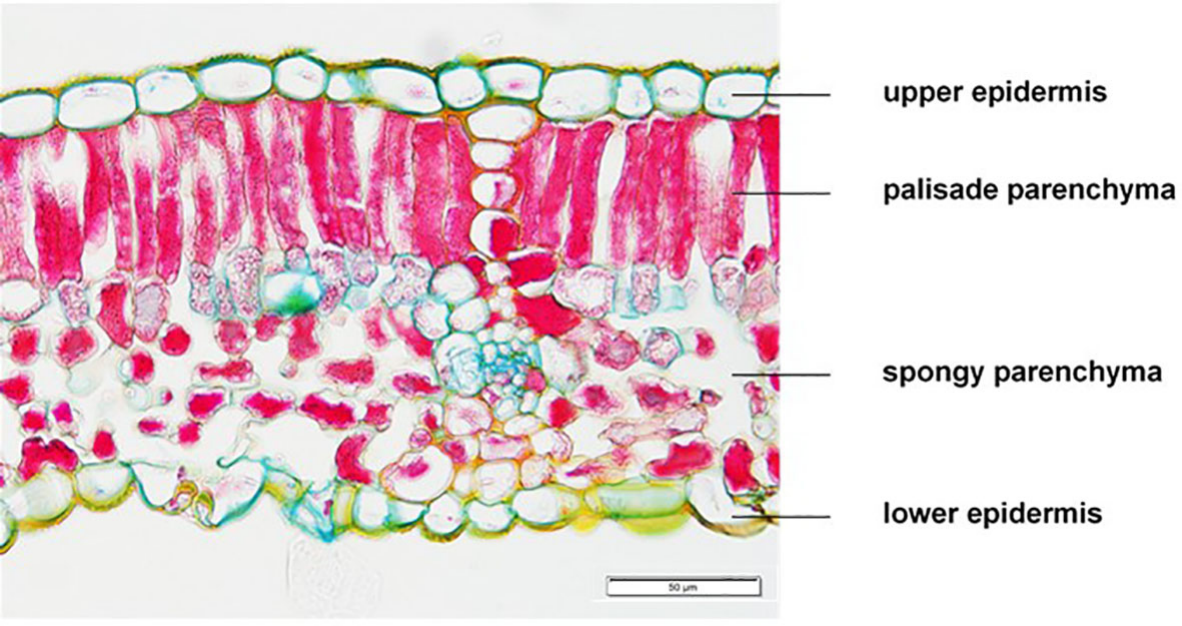
Histological tissue section of a *Vitis vinifera* cv. Riesling leaf as basis for analysis of epidermal and parenchymatic shares.

For morphological traits six leaves per repetition of each CO_2_ treatment were sampled on the same dates in 2015 (02/09) and 2016 (30/08). Cut leaves were rolled and immediately fixed in tubes containing a FAA solution (70% ethanol, 20% H_2_O, 5% formaldehyde and 5% glacial acetic acid). After 24 h leaf samples were transferred and stored in tubes with an 70% ethanol solution until further processing. Later, rolled leaves were cut in slices following dehydration by using an increasing ethanol/isopropanol series, infiltration and embedding in paraffin under low air pressure conditions. By using a rotary microtome (Leica, RM 2155, Nussloch, Germany) sections of 5 µm were prepared and fixed on microscopic slides. Then, the sections were triple stained after the W3A method according to Wacker (2006) by using acridine red CI45000, acriflavin CI46000 and astral blue CI48048 in combination with ethanol, dest. water and glacial acetic acid following washing and differentiation with isopropanol. Pictures of the leaf cross-sections were taken using a fluorescence microscope (Keyence, Biozero BZ-8000K, Neu-Isenburg, Germany). Measurements of pictures were conducted with ImageJ, an image analysis software (National Institutes of Health, Bethesda, MD, USA). Then, thickness of the upper and lower epidermis, the palisade and sponchy parenchyma were recorded (**Figure 1**). Pictures published in this work were taken with an additional microscope (Mikroskop BX53 Olympus Deutschland GmbH).

### Statistical analyses

Statistical analyses were performed with the statistical software R, version 3.4.2 (R Foundation for Statistical Computing, Vienna, Austria). Data for all parameters were tested using multi-factor (treatment, block, year and interaction treatment x year as well as treatment x date) analysis of variance (ANOVA) and Tukey’s honestly significant difference (HSD) test for significant differences (*P* ≤ 0.05 level). For all parameters, means per ring were calculated and used for statistical analyses.

## Results

**Figure 2:**
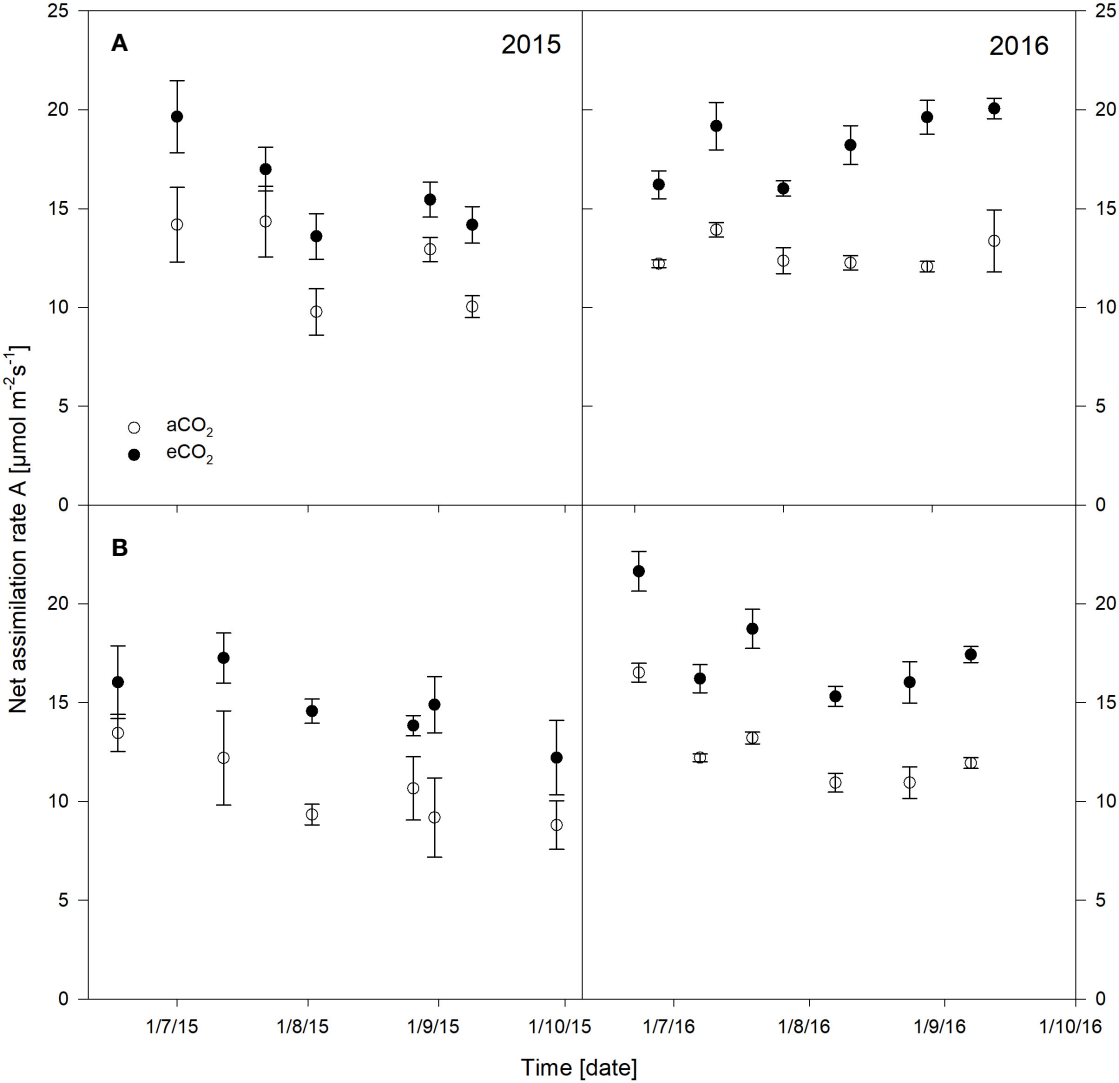
Net assimilation rate of *Vitis vinifera* cvs. Cabernet Sauvignon **(A)** and Riesling **(B)** measured over the seasons 2015 and 2016 under aCO_2_ and eCO_2_ conditions. Data represent mean ± SD of the three rings and nine leaves per treatment.

The net assimilation rates were significantly stimulated under eCO_2_ for both cultivars and seasons, which are presented in **Figure 2** and have previously been described for stomatal conductance, water use efficiency, pre-dawn leaf water potential as well as for pruning weight or leaf area (Wohlfahrt et al., 2018). Additionally, results of the statistical output are shown in **Table 1**. Cabernet Sauvignon net assimilation rate increased from 18% up to 41% in 2015 under eCO_2_ conditions, and showed +31% on a seasonal average. In 2016, the increase was 25% up to 63% with an average of +42% under eCO_2_. Net assimilation of Riesling was 19% to 62% higher under eCO_2_ in 2015 showing a seasonal average of a 41% increase. The gain in 2016 ranged between 31% to 46% with a seasonal average of +40%. Overall, Riesling was stimulated higher in net assimilation under eCO_2_ in 2015, whereas in 2016 cultivars did not differ in their rate of increase (approx. 40%). It was obvious that in both cultivars the year as well as the measuring date have to be considered as independent factors. For Cabernet Sauvignon an interaction between the treatment and year and date occurred (**Table 1**).

**Table 1:** Results of the multi-factor analysis of variance (ANOVA) and Tukey’s honestly significant difference (HSD) test for net assimilation of the two cultivars Riesling (R); Cabernet Sauvignon (CS) over the two seasons and measuring dates. Significant differences appear at *P* ≤ 0.05 level and are displayed in bold type.

	*P* value	
	R	CS
*treatment*	***2.2-e16***	***2.2-e16***
*block*	*0.3130*	*0.8637*
*year*	***1.365e-11***	***5.522e-06***
*date*	***1.041e-12***	***4.374e-11***
*treatment x year*	*0.1804*	***0.0012***
*treatment x date*	*0.3289*	***0.0156***

Optical leaf clip meter indices did not differ between treatments or years for both cultivars (**Table 2**). Only for Riesling a trend to higher Chl index under CO_2_ enrichment over the two years (*P=0.0629*) was observed. Whereas NBI index was higher, Flav index was lower in leaves of Cabernet Sauvignon compared to Riesling in both years. As shown in **Table 2**, leaf pigments (Chl a, Chl b, total Chl and Car) were affected by the year and not by eCO_2_.

**Table 2:** Results of optical leaf clip meter readings of leaf chlorophyll (Chl), flavonols (Flav) and nitrogen balance index (NBI) as well as leaf pigment content (in dry matter, DM) for chlorophyll a (Chl a), chlorophyll b (Chl b), total chlorophyll (Chl total) and carotenoid (Car) of the two cultivars Riesling (R) and Cabernet Sauvignon (CS) under aCO_2_ and eCO_2_ conditions.

	Dualex indices			mg g^-1^ DM			
	Chl	Flav	NBI	Chl a	Chl b	Chl total	Car
2015							
R aCO_2_	26.09 ± 2.26	2.90 ± 0.04	9.02 ± 0.68	2.16 ± 0.28	1.04 ± 0.16	2.67 ± 0.36	0.64 ± 0.07
R eCO_2_	29.11 ± 2.63	2.92 ± 0.04	10.04 ± 0.76	2.16 ± 0.29	1.07 ± 0.14	2.68 ± 0.35	0.69 ± 0.09
2016							
R aCO_2_	27.02 ± 3.04	2.80 ± 0.07	9.75 ± 1.31	3.95 ± 0.30	3.58 ± 0.27	5.70 ± 0.39	0.94 ± 0.13
R eCO_2_	30.31 ± 1.06	2.86 ± 0.11	10.65 ± 0.57	3.81 ± 0.31	3.43 ± 0.18	5.48 ± 0.40	0.93 ± 0.10
*P* value							
*treatment*	*0.0629*	*0.3883*	*0.1152*	*0.8264*	*0.7360*	*0.7966*	*0.6282*
*block*	*0.5896*	*0.8216*	*0.6142*	*0.3345*	*0.1627*	*0.2655*	*0.5203*
*year*	*0.4799*	*0.1030*	*0.2499*	***1.897e-05***	***5.658e-08***	***2.306e-06***	***0.0023***
*treatment x year*	*0.9256*	*0.7516*	*0.9143*	*0.8375*	*0.4907*	*0.7397*	*0.7439*
2015							
CS aCO_2_	29.66 ± 3.52	2.64 ± 0.09	11.32 ± 1.58	2.09 ± 0.31	0.98 ± 0.15	2.58 ± 0.38	0.60 ± 0.10
CS eCO_2_	29.54 ± 2.81	2.62 ± 0.21	11.50 ± 1.99	2.41 ± 0.28	1.17 ± 0.15	2.98 ± 0.35	0.70 ± 0.08
2016							
CS aCO_2_	29.03 ± 3.07	2.53 ± 0.11	11.63 ± 1.68	4.22 ± 0.23	3.73 ± 0.11	6.04 ± 0.28	0.96 ± 0.11
CS eCO_2_	31.41 ± 1.63	2.58 ± 0.07	12.28 ± 0.99	4.54 ± 0.48	3.71 ± 0.08	6.36 ± 0.48	1.11 ± 0.21
*P* value							
*treatment*	*0.4835*	*0.8502*	*0.6456*	*0.1576*	*0.3410*	*0.1603*	*0.1748*
*block*	*0.1780*	*0.3764*	*0.1891*	*0.2723*	*0.5223*	*0.2817*	*0.2614*
*year*	*0.6995*	*0.3339*	*0.5484*	***1.055e-05***	***3.584e-09***	***9.703e-07***	***0.0016***
*treatment x year*	*0.4388*	*0.6339*	*0.7930*	*0.9500*	*0.1701*	*0.7615*	*0.8164*

Data represent mean ± SD of the three rings and six leaves per treatment. Tukey’s honestly significant difference (HSD) test for significant differences appear at *P* ≤ 0.05 level and are displayed in bold type.

**Figure 3:**
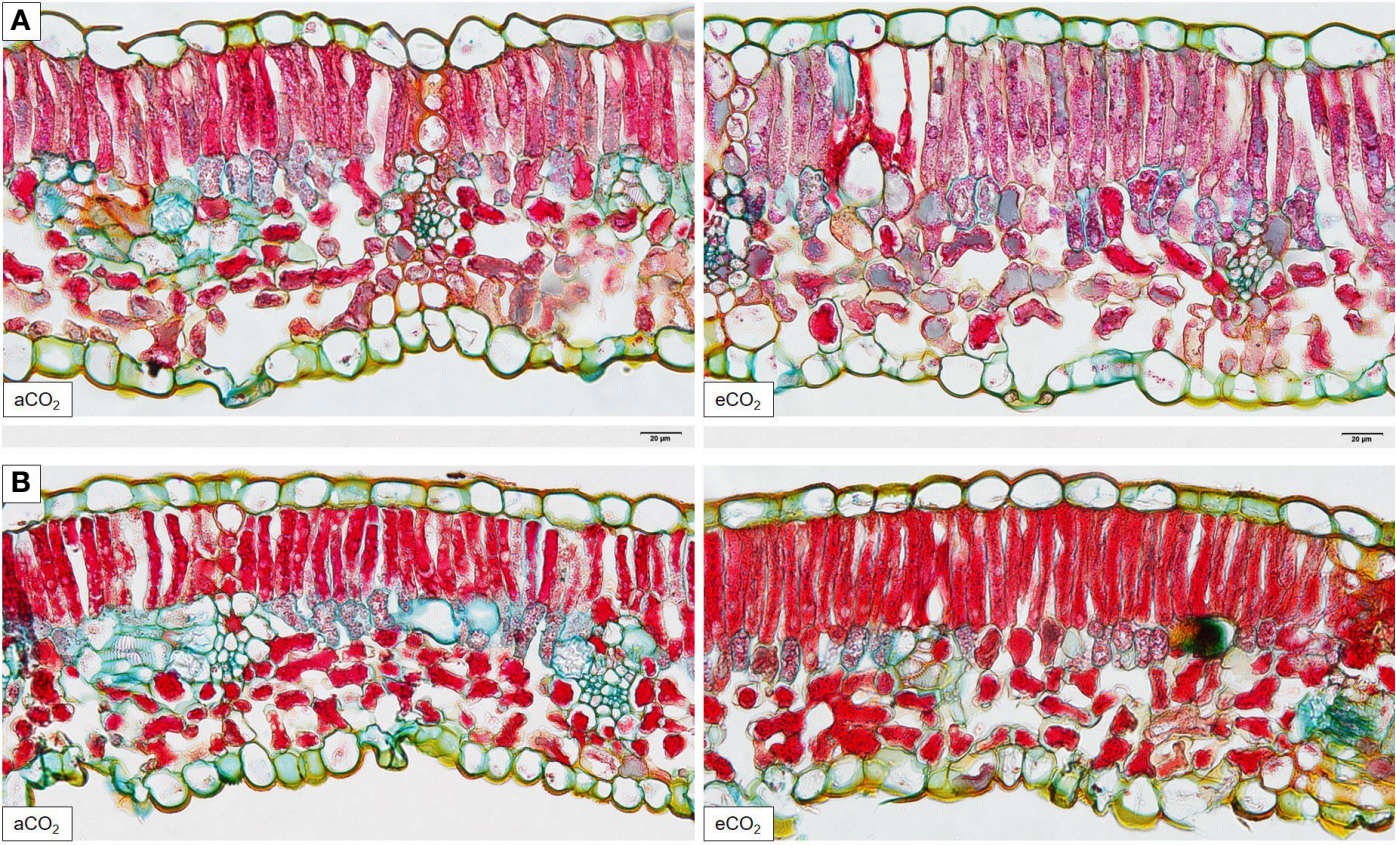
Histological analysis of aCO_2_ and eCO_2_ leaf cross-sections of *Vitis vinifera* cvs. Cabernet Sauvignon **(A)** and Riesling **(B)** stained with W3A (20 μm, 400x).

Total leaf thickness and width of spongy parenchyma of Cabernet Sauvignon (**Figure 3A**) and Riesling (**Figure 3B**) remained less affected under eCO_2_ conditions (**Table 3**). However, significant differences were found in histological analyses of the leaf cross-sections between the two CO_2_ treatments in upper and lower epidermis and the palisade parenchyma of Cabernet Sauvignon (**Figure 3A**). Whereas under eCO_2_ the palisade parenchyma increased, the epidermal tissue decreased in thickness. Also, palisade parenchyma in Riesling showed a trend in increase under eCO_2_ (**Table 3**), no significance difference was detected. However, the ratio between palisade and spongy parenchyma hardly differed between the CO_2_ treatments in Riesling whilst in Cabernet Sauvignon the treatment effect was significantly pronounced (*P=0.017*) with an increasing ratio under eCO_2_. Leaf layer thickness of both cultivars was affected by the year, like the epidermis and palisade parenchyma, the latter appeared to have higher values in 2015 (**Table 3**). Additionally, total leaf thickness and ratio between palisade and spongy parenchyma showed an effect by the year in Cabernet Sauvignon. Block effects occurred for both cultivars in total thickness of the leaf and the spongy parenchyma thickness.

**Table 3:** Thickness of total leaf tissue, palisade parenchyma, spongy parenchyma and ratio of palisade to spongy parenchyma of the two cultivars Riesling (R) and Cabernet Sauvignon (CS) under aCO_2_ and eCO_2_ conditions.

	Thickness [µm]				
	total thickness	upper/lower epidermis	palisade parenchyma	spongy parenchyma	palisade/spongy parenchyma ratio
2015					
R aCO_2_	171.98 ± 5.71	34.82 ± 3.09	51.65 ± 4.08	86.64 ± 1.61	0.61 ± 0.04
R eCO_2_	177.35 ± 4.93	39.75 ± 1.28	55.60 ± 4.74	83.15 ± 6.16	0.69 ± 0.11
2016					
R aCO_2_	166.89 ± 14.30	34.40 ± 2.71	45.79 ± 1.97	83.48 ± 9.15	0.56 ± 0.06
R eCO_2_	176.22 ± 14.62	33.98 ± 1.90	50.37 ± 4.09	87.35 ± 11.98	0.58 ± 0.04
*P* value					
*treatment*	*0.1528*	*0.0954*	*0.0937*	*0.9553*	*0.1987*
*block*	***0.0250***	*0.0901*	*0.3021*	***0.0169***	*0.2110*
*year*	*0.5189*	***0.0329***	***0.0396***	*0.8780*	*0.0687*
*treatment x year*	*0.6794*	*0.0555*	*0.8892*	*0.2980*	*0.4998*
2015					
CS aCO_2_	191.99 ± 7.58	40.05 ± 5.99	59.40 ± 2.85	89.52 ± 3.18	0.68 ± 0.03
CS eCO_2_	195.97 ± 9.87	34.78 ± 1.35	66.66 ± 3.97	93.91 ± 4.62	0.72 ± 0.00
2016					
CS aCO_2_	172.37 ± 10.80	34.78 ± 0.40	48.88 ± 4.30	87.19 ± 7.97	0.56 ± 0.01
CS eCO_2_	175.00 ± 11.42	31.20 ± 3.17	56.00 ± 0.38	85.42 ± 10.07	0.67 ± 0.08
*P* value					
*treatment*	*0.3301*	***0.0405***	***0.0039***	*0.6341*	***0.0170***
*block*	***0.0030***	*0.1136*	*0.1404*	***0.0107***	*0.0960*
*year*	***0.0004***	***0.0403***	***0.0004***	*0.0786*	***0.0080***
*treatment x year*	*0.8367*	*0.6470*	*0.9689*	*0.2801*	*0.1764*

Data represent mean ± SD of the three rings and six leaves per treatment. Tukey’s honestly significant difference (HSD) test for significant differences appear at *P* ≤ 0.05 level and are displayed in bold type.

## Discussion

Responses of two different grapevine cultivars grown in the VineyardFACE-system indicate that an increase in atmospheric CO_2_ predicted for the mid-century affects leaf gas exchange, and especially enhances net assimilation. This is in accordance with results obtained from previous studies on field-grown grapevines (Bindi et al., 2001; Moutinho-Pereira et al., 2009; Edwards et al., 2017) and a multitude of other C_3_ crop species under elevated CO_2_ concentrations. In a previous VineyardFACE trial both, Riesling and Cabernet Sauvignon had frequently higher photosynthetic rates in their early years of adaptation and increased in leaf as well as fruit biomass production. Hence, an impact on single berry weight, cluster weight and bunch architecture has been shown (Wohlfahrt et al., 2018 and Wohlfahrt et al., 2020). Even though net assimilation was highly stimulated for both cultivars under a relative low CO_2_ increase (+39% net assimilation vs. +20% CO_2_ increase), no impact was found in chlorophyll content nor lead to changes in other leaf pigments or leaf nitrogen status.

That the NBI index in leaves differs within different grapevine cultivars and that Chl index is used as indicator for leaf nitrogen content was reported by Cerovic et al. (2015), and could further provide information about the nutrition status of berries. Interestingly, the differences found between the leaves of the two cultivars for Chl index and NBI were also detected earlier during berry ripening in 2015 and 2016 by higher amino acid concentration in berries of Cabernet Sauvignon in comparison to Riesling (Wohlfahrt et al., 2020). These cultivar dependent differences, probably influenced by the choice of rootstock and the scion-rootstock combination as well, were found for various plant growth parameters, e.g. lateral leaf area or perennial wood growth (Wohlfahrt et al., 2018). Differences in leaf nutrition status by using optical leaf clip meter indices or leaf pigment content have not been found between the two CO_2_ treatments and for neither of the two cultivars, which corroborates the results of Moutinho-Pereira et al. (2009) when using a SPAD meter. Leaf nitrogen status relates to the photosynthetic capacity and that is why leaves form the highest growth demand for nitrogen (Evans, 1989), while under elevated CO_2_ leaf nitrogen content generally decreases by a N-dilution effect caused by the increase in carbohydrate accumulation through enhanced net assimilation (Feng et al., 2015). Thus, it remains unclear if the two cultivars within the VineyardFACE will decrease in leaf nitrogen under eCO_2_ in future as variations in nitrogen content are also depending on the initial nitrogen limitation status of the single plant (Stitt and Krapp, 1999; Ainsworth and Long, 2005).

Leaf pigments (Chl a, Chl b, total Chl and Car) were not altered under eCO_2_ which is in accordance with results of total chlorophyll and carotenoid content in beech leaves, where eCO_2_ revealed no effects (Polle et al., 1997). Only a varying nutrient supply caused significant differences in leaf pigments of beech. The seasonal differences in leaf pigments shown for both cultivars were expected due to their dependence on environmental factors such as water availability (Fanizza et al., 1991), which differed in rainfall 2015 (230 mm) and 2016 (369 mm) during growing season. Leaf chlorophyll pigments (Chl a, Chl b, Chl total) were reduced about 50% and carotenoids by 30% in 2015, when precipitation was shortened in comparison to 2016.

Histological analyses of the grapevine leaf cross-sections revealed no increase in total leaf thickness under elevated CO_2_. Other C_3_ species, particularly soybean, loblolly pine and sweet gum showed an increase in leaf thickness under different CO_2_ enrichment scenarios (Thomas and Harvey, 1983), and in different poplar clones in the early phase of growth (Radoglou and Jarvis, 1990). Furthermore, leaves of crop species were reported to exhibit greater increases in leaf thickness compared to wild species (Pritchard et al., 1999), but in this review only experiments conducted in chambers (growth chamber and open top chamber), glass houses and phytotrons have been considered. However, effects of elevated CO_2_ on leaf anatomy were summarized to depend on leaf development stage, soil fertility, and again, season of the year (Pritchard et al., 1999). The latter is in accordance with the total thickness of epidermis and palisade parenchyma of Riesling and Cabernet Sauvignon, which were enlarged in 2015 compared to 2016 and thus affected by the season. The differences in leaf thickness could be attributed to extreme temperatures in the growing season 2015 (29 heat days (≥30°C) compared to 17 heat days in 2016) since under high temperature conditions an increase in thickness of grapevine leaves was reported (Salem-Fnayou et al., 2011). Still, both types of ground tissue, palisade and spongy parenchyma contain chloroplasts. Even though the palisade parenchyma contains a high number of chloroplasts compared to the spongy parenchyma, the latter is very prominent in terms of the intercellular air space in the lower mesophyll. Chlorenchyma and aerenchyma are both of utmost importance for the photosynthetic rate which in parts may help to explain that under eCO_2_ the photosynthetic activity will be further stimulated, since a higher internal leaf surface enhances the ability to absorb CO_2_ to a larger extent. In a previous study on grapevines (cv. Touriga Franca) under open top chamber conditions authors assumed that an increased leaf and therefore parenchyma thickness under eCO_2_ was due to an enlargement of cells rather than increased cell division (Moutinho-Pereira et al., 2009), which was previously suggested by Pritchard et al. (1999). This could be explained by the same amount of parenchyma layers in both CO_2_ treatments (**Figure 3**, data not shown). Nevertheless, thickness of palisade parenchyma increased, at least for Cabernet Sauvignon under eCO_2_. These morphological alterations of leaf layers and extension of cells under eCO_2_ were found in other agricultural C_3_ species (Thomas and Harvey, 1983; Robertson and Leech, 1995). Surprisingly, instead of an expansion in leaf thickness Cabernet Sauvignon epidermal thickness decreased under higher CO_2_ concentration. That an increase in leaf tissues within the mesophyll happens at the expense of epidermis (Garnier et al., 1999), and could therefore lead to increasing foliage photosynthetic potentials was proposed by Niinemets (1999) and approved in this study. Contrary to the leaf morphology of the red cultivar Touriga Franca, which resulted in thicker spongy parenchyma and thus lower or unchanged palisade to spongy parenchyma ratio (Moutinho-Pereira et al., 2009), the palisade to spongy parenchyma ratio increased under eCO_2_ within Cabernet Sauvignon under open field conditions. This leads to the assumption that chamber experiments are not fundamentally comparable with studies conducted under field conditions on the one hand, and cultivar specific leaf characteristics and responses on the other hand (Boso et al., 2010). Also, different ‘climatic’ effects are possibly responsible for the differences in parenchyma responses. In addition, Riesling (cool to intermediate) and Cabernet Sauvignon (warm) belong to different climate maturity groupings based on average growing season temperatures (Jones et al., 2005). Under these requirements, different plant reaction of the two cultivars are expected with the accessory climatic changes apparent from season to season which were recently shown (Wohlfahrt et al., 2018). In a study based on climate and developmental plasticity with regards to the seasonal variability in grapevine leaf morphology, results demonstrated that besides environmental, genetic and developmental effects influence the leaf shape in a way largely independent of each other (Chitwood et al., 2016).

Eventually, free air CO_2_ enrichment studies are essential to understand plant responses to a changing climate, especially for permanent plant crops and obtained results are likely to improve the current understanding of physiological and structural responses of plants to future environmental conditions, e.g. elevated CO_2_ levels.

## Conclusion

Results observed on leaf physiology and morphological characteristics of cvs. Riesling and Cabernet Sauvignon can provide first insights to seasonal adaptation strategies of grapevines under a changing climate and in particular to future elevated CO_2_ concentrations. However, regardless of the CO_2_ treatment the effect of the season and in particularly high temperature and low precipitation can modify the plant response to eCO_2_. Thus, the plant water as well as nutrition status may have a large impact on leaf morphology too. For these reasons, field studies on the effect of elevated CO_2_, especially by using non-herbaceous perennial plants, are complex and difficult to execute and thus need a long-term investigation over at least two decades. Therefore, studies like the present one are welcome to improve our knowledge about the response of plants to future environmental conditions under realistic conditions.

Furthermore, as the plant nutrient status is suggested to be linked to the antioxidative enzyme response under elevated CO_2_ concentrations (Schwanz et al., 1996) the nutritional status of the leaves and the whole plant needs to be intensified in further VineyardFACE studies. In addition, investigations should be carried out in the direction of carbon sink and in regards to the C/N ratio in the soil if it is assumed that a higher surface litter input due to more leaf biomass under eCO_2_ could also stimulate the rate of mineralization.

## Data availability statement

The original contributions presented in the study are included in the article/**Supplementary Material**, further inquiries can be directed to the corresponding author/s.

## Author contributions

Conceptualization: YW. Methodology: YW, KK, DP, and ST. Formal analysis: YW. Investigation: YW and ST. Resources: MS. Data curation: YW and ST. Writing—original draft preparation: YW, KK, DP, ST, and MS. Writing—review and editing: YW and MS. Visualization: YW, KK, and DP. Supervision: MS. Project administration: YW, ST, and MS. Correspondence with the journal’s editor: YW. All authors contributed to the article and approved the submitted version.
