# Supplementary material for: Grapevine leaf physiology and morphological characteristics to elevated CO2 in the VineyardFACE (Free air Carbon dioxide Enrichment) experiment
Source: Front Plant Sci. 2022 Dec 9;13:1085878. doi: 10.3389/fpls.2022.1085878 (PMC9782973; doi:10.3389/fpls.2022.1085878)
Supplement: Supplementary file 1 [file DataSheet_1.docx]

Supplementary Material


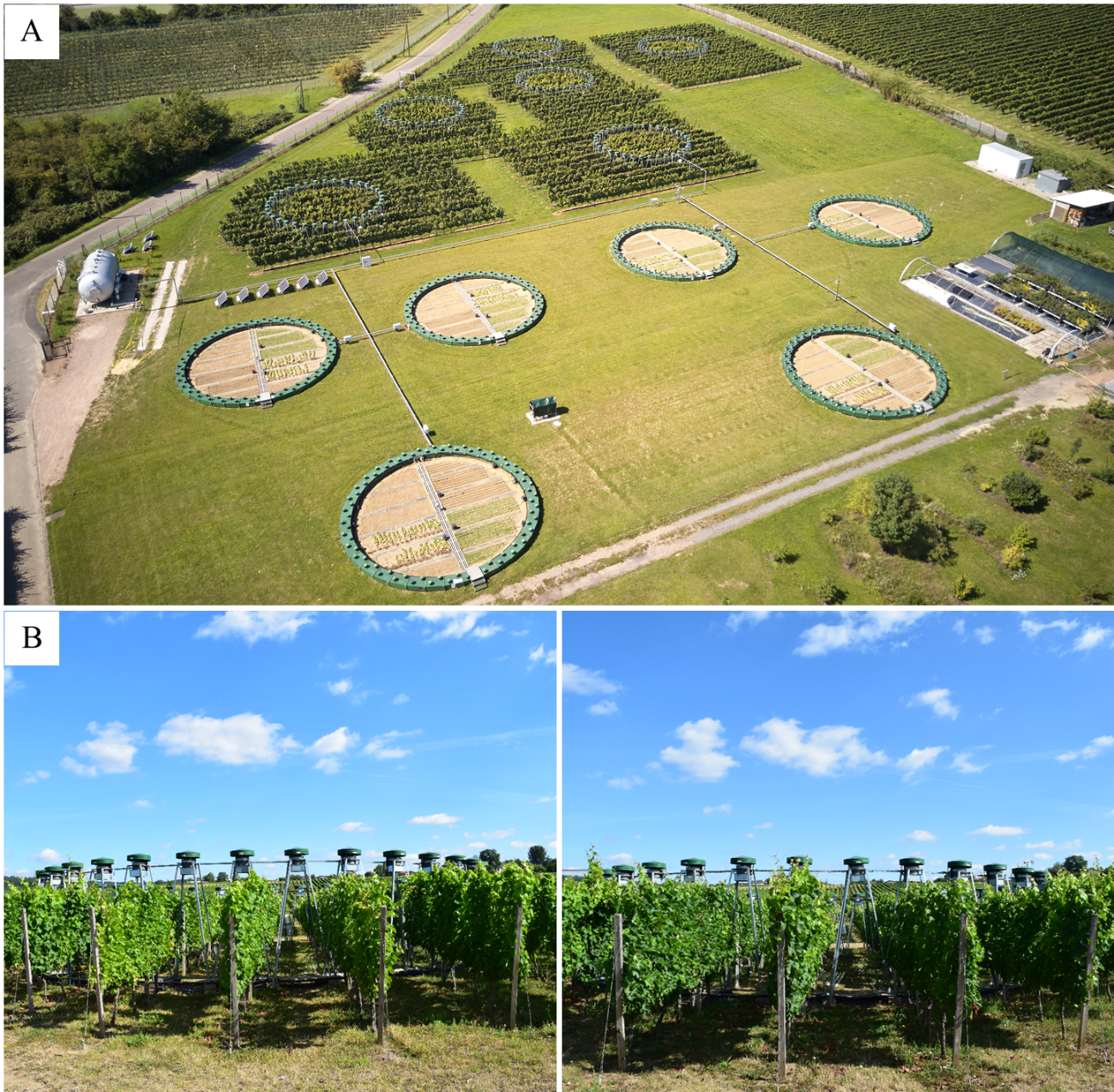


**Supplementary Figure 1.** Examples of FACE systems with the typical ring structure. A. Two FACE systems as part of the special crop FACE system at Geisenheim University, Rheingau, Germany. The VineyardFACE as example of a traditional-regional permanent crop (top) and the VegetableFACE as example of different special annual crops (bottom). © Winfried Schönbach. B. Control ring (left) and CO_2_-enriched ring (right) of the VineyardFACE system with two grapevine cultivars (*Vitis vinifera*).


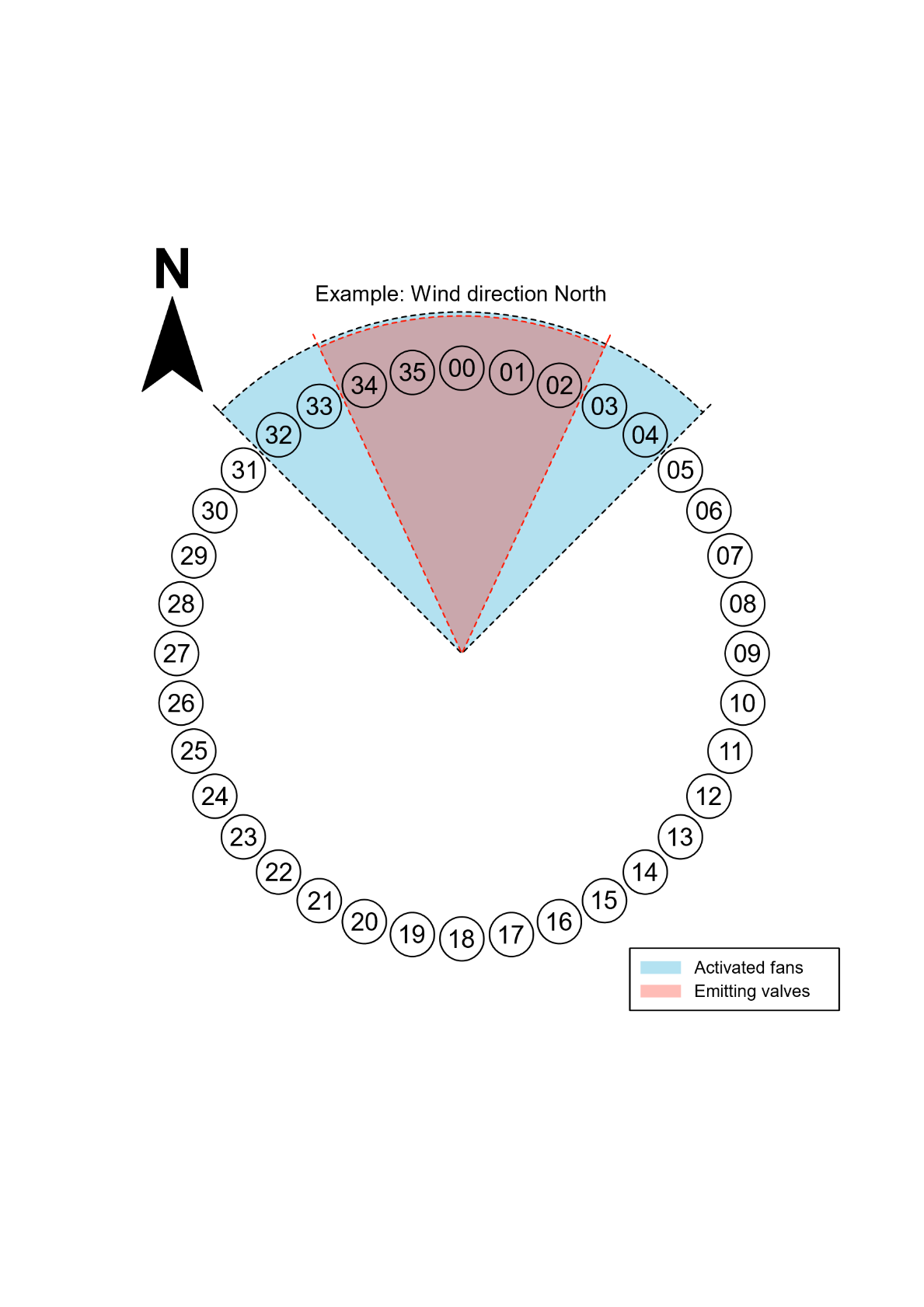


**Supplementary Figure 2.** Schematic overview of the fan and valve assembly within a FACE ring. During wind direction north the nine fans 32, 33, 34, 35, 00, 01, 02, 03, and 04 were activated but only the valves in 34, 35, 00, 01, and 02 were emitting CO_2_.


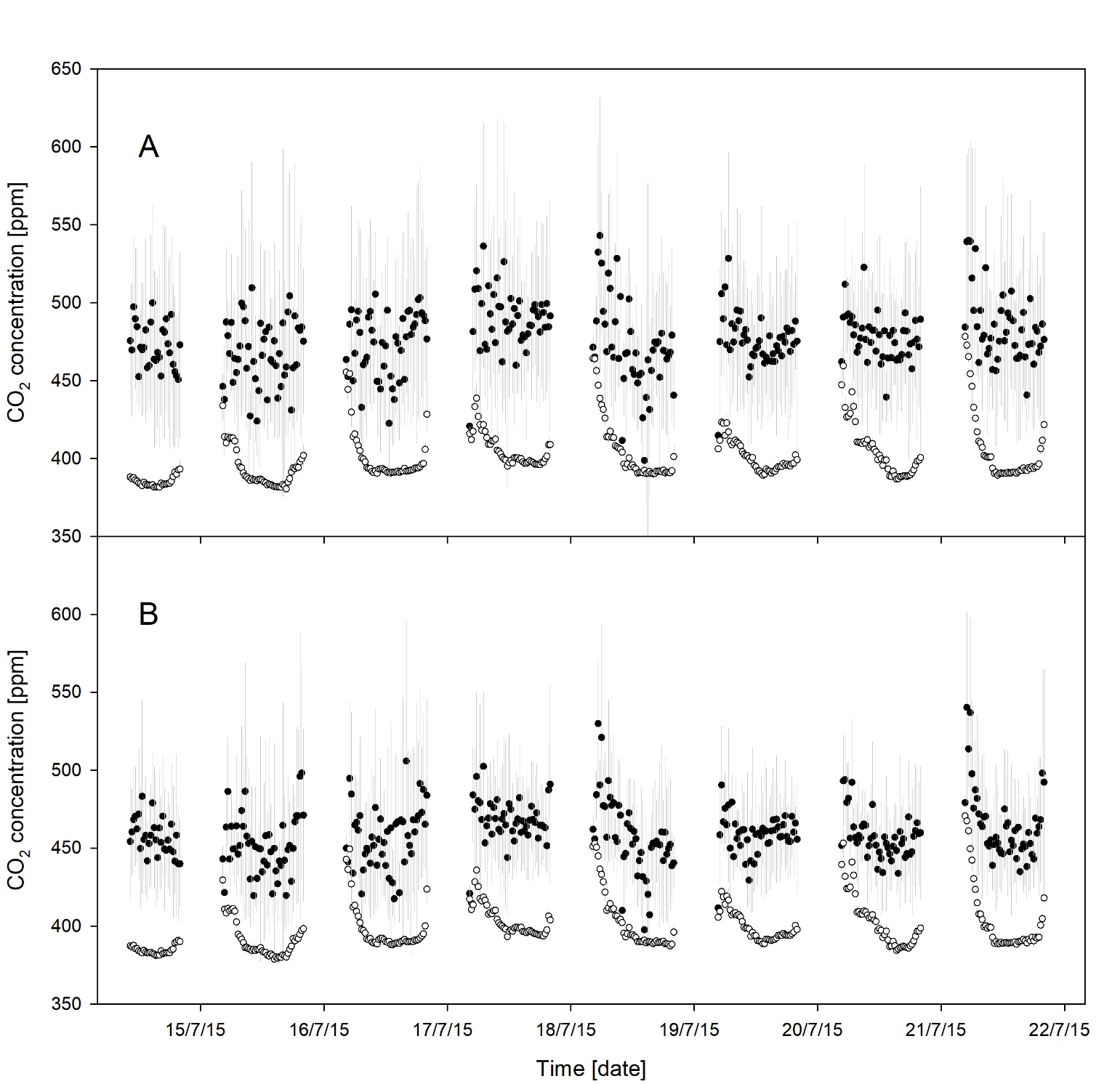


**Supplementary Figure 3.** Daily averaged carbon dioxide concentration of aCO_2_ (open circles) and eCO_2_ (filled circles) treatments measured from 14-22/07/2015 at heights of 0.8 m (A) and 1.7 m (B) from sunrise to sunset at the VineyardFACE, Geisenheim. Means ± sd.


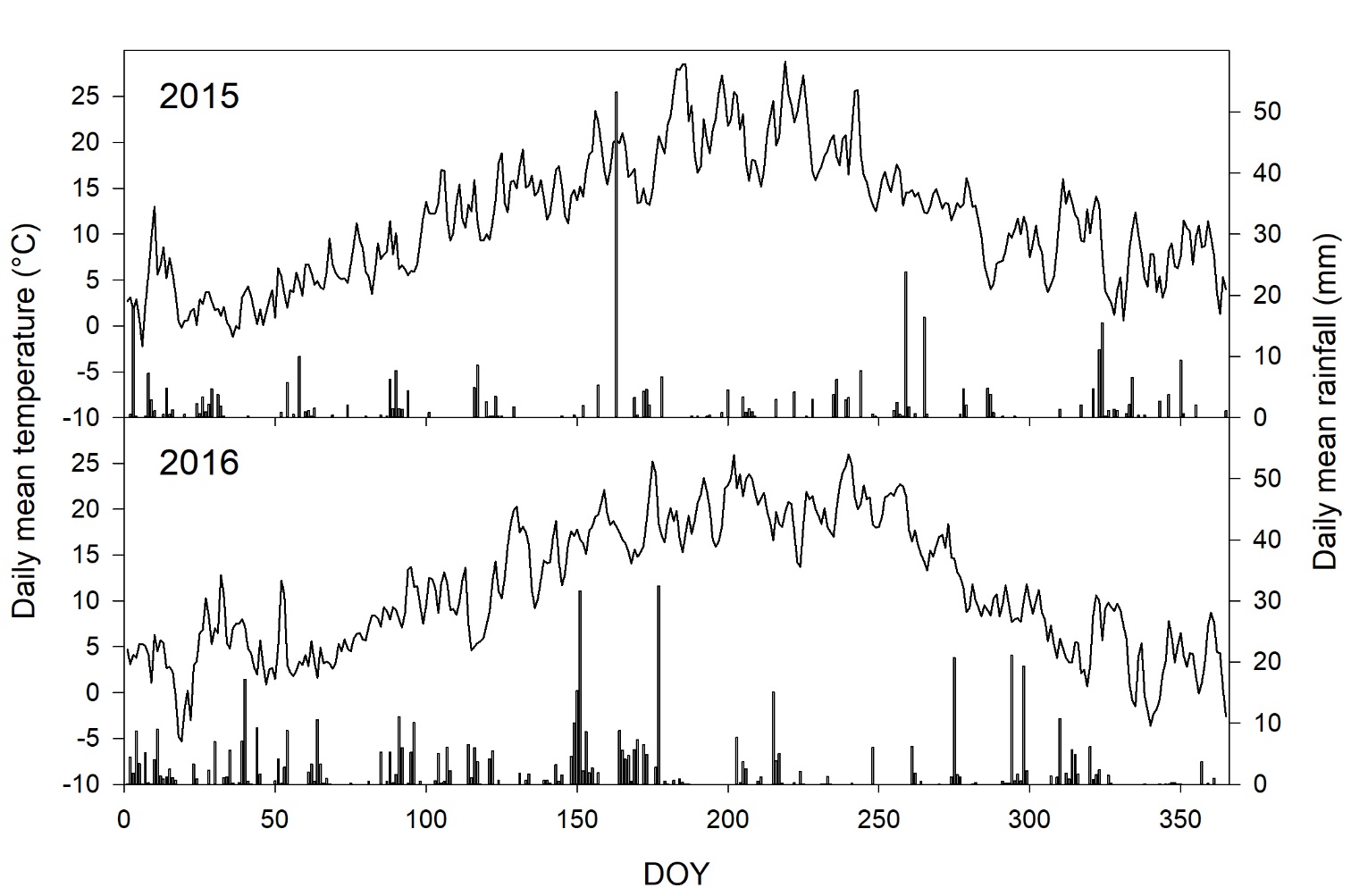


**Supplementary Figure 4.** Daily course of mean air temperature (solid line) and rainfall (black bars) in 2015 and 2016 at the VineyardFACE site, Geisenheim. DOY = day of year.
